# Over Their Graves

**Published:** 1889-10

**Authors:** Henry Jerome Stbckard


### OVER THEIR GRAVES.

OVER their graves rang once the bugle's call,
The searching shrapnel, and the crashing ball;
The shriek, the shock of battle, and the neigh
Of horse; the cries of anguish and dismay;
And the loud cannon's thunders that appall.

Now through the years the brown pine-needles fall,
The vines run riot by the old stone wall,
By hedge, by meadow streamlet, far away,
Over their graves!

We love our dead wher'er so held in thrall,—
Than they no Greek more bravely died, nor Gaul,—
A love that's deathless! but they look to-day
With no reproaches on us when we say,
"Come! let us clasp your hands, we're brothers all,"
Over their graves!

—*Henry Jerome Stbckard*, in THE CENTURY.

